# Redox and Antioxidant Modulation of Circadian Rhythms: Effects of Nitroxyl, N-Acetylcysteine and Glutathione

**DOI:** 10.3390/molecules26092514

**Published:** 2021-04-26

**Authors:** Santiago Andrés Plano, Fernando Martín Baidanoff, Laura Lucía Trebucq, Sebastián Ángel Suarez, Fabio Doctorovich, Diego Andrés Golombek, Juan José Chiesa

**Affiliations:** 1 Institute for Biomedical Research (BIOMED), Catholic University of Argentina (UCA) and National Scientific and Technical Research Council (CONICET), C1107CABA Buenos Aires, Argentina; santiagoplano@uca.edu.ar; 2 Laboratorio de Cronobiología, Departamento de Ciencia y Tecnología, Universidad Nacional de Quilmes (UNQ), Consejo Nacional de Investigaciones Científicas y Técnicas (CONICET), Roque Sáenz Peña 352, B1876BXD Bernal, Argentina; fbaidanoff@gmail.com (F.M.B.); laura.trebucq@gmail.com (L.L.T.); juan.chiesa@unq.edu.ar (J.J.C.); 3 Departamento de Química Inorgánica, Analítica y Química Física/INQUIMAE-CONICET, Facultad de Ciencias Exactas y Naturales, Universidad de Buenos Aires, Ciudad Universitaria, Pab. II, C1428EHA Buenos Aires, Argentina; seba@qi.fcen.uba.ar (S.Á.S.); fabio.doctorovich@gmail.com (F.D.)

**Keywords:** suprachiasmatic nuclei, cGMP, Angeli’s salt, reactive nitrogen species

## Abstract

The circadian clock at the hypothalamic suprachiasmatic nucleus (SCN) entrains output rhythms to 24-h light cycles. To entrain by phase-advances, light signaling at the end of subjective night (circadian time 18, CT18) requires free radical nitric oxide (NO•) binding to soluble guanylate cyclase (sGC) heme group, activating the cyclic guanosine monophosphate (cGMP)-dependent protein kinase (PKG). Phase-delays at CT14 seem to be independent of NO•, whose redox-related species were yet to be investigated. Here, the one-electron reduction of NO• nitroxyl was pharmacologically delivered by Angeli’s salt (AS) donor to assess its modulation on phase-resetting of locomotor rhythms in hamsters. Intracerebroventricular AS generated nitroxyl at the SCN, promoting phase-delays at CT14, but potentiated light-induced phase-advances at CT18. Glutathione/glutathione disulfide (GSH/GSSG) couple measured in SCN homogenates showed higher values at CT14 (i.e., more reduced) than at CT18 (oxidized). In addition, administration of antioxidants N-acetylcysteine (NAC) and GSH induced delays per se at CT14 but did not affect light-induced advances at CT18. Thus, the relative of NO• nitroxyl generates phase-delays in a reductive SCN environment, while an oxidative favors photic-advances. These data suggest that circadian phase-locking mechanisms should include redox SCN environment, generating relatives of NO•, as well as coupling with the molecular oscillator.

## 1. Introduction

The mammalian circadian clock at the hypothalamic suprachiasmatic nuclei (SCN) orchestrates daily rhythms in a broad range of behavioral and physiological processes, driving autonomic and endocrine outputs which coordinate a network of oscillators in peripheral tissues [1]. This is achieved by the entrainment (i.e., phase-shifting) of the SCN oscillator activity to equal the 24-h light-dark cycle. Interacting transcriptional-translational feedback loops (TTFLs) of clock genes generate core oscillator activity at the SCN cells and most peripheral tissues. A transcriptional activator, formed by circadian locomotor output cycles kaput (CLOCK) and brain and muscle aryl hydrocarbon receptor nuclear translocator-like protein 1 (BMAL1) heterodimer, binds to enhancer elements at the promoter of repressor clock genes *period (per) 1, 2*, and *3*, and *cryptochrome (cry) 1* and *2*, increasing their transcriptional activity. In turn, PERs and CRYs proteins accumulate in the cytoplasm and heterodimerize, generating transcriptional repressors by interfering with the CLOCK:BMAL heterodimers, closing the cycle each 24 h. Entrainment of the SCN clock to the light-dark cycles involves the communication of photic information through several neurotransmitters and signal transduction pathways, whose endpoint effectors converge to modify the activity of clock genes at the transcriptional level [2]. Moreover, the TTFL circadian clockwork might be modulated by the redox state. Indeed, several redox pairs have been shown to interact with either circadian clock proteins or transcriptional regulators in vitro [3,4]. The SCN, NADP+/NADPH, FAD+/FADH, and dehydroascorbic acid/ascorbic acid redox couples, as well as the global protein glutathiolation, exhibit circadian oscillations, while the resting electrical activity of SCN neurons is also sensitive to oxidants and reductants [5].

Brief light stimulation during the circadian night (i.e., the time of behavioral activity in nocturnal rodents) generates a phase-shift of the SCN clock, and thereby its behavioral and physiological rhythmic outputs [6]. The specific circadian time (CT) at which the light pulse is applied is determinant of whether phase-delays or advances are induced. At CT14 (i.e., 2 h after locomotor activity onset, CT12), a phase-delay of locomotor rhythm is generated, while phase-advances occur after stimulation at the late circadian night at CT18. Phase-shifts are transduced by the glutamatergic influx through efferents from the retinal ganglion cells to ventral SCN neurons, which promotes calcium influx through glutamate-N-methyl-D-aspartate receptors (NMDAr) and triggers calcium-dependent calmodulin-kinase phosphorylation of the nitric oxide synthase (nNOS), increasing its activity [7]. Downstream, the pathway for advances proceeds through NO• coordination of regulatory ferrous heme on the soluble guanylyl cyclase (sGC), increasing cGMP synthesis and, subsequently, the cGMP-dependent protein kinase (PKGII) activity [8] to phosphorylate its dependent substrate [9]. At CT14, circadian phase-delays proceed through other pathways (e.g., adenylate cyclase/cyclic adenosine monophosphate (cAMP)/cAMP-activated protein kinase (PKA)) [2] activated by calcium influx through the aperture of endoplasmic ryanodine type 2 receptor channels [10]. Downstream, both pathways converge at cAMP-responding element-binding protein (CREB) phosphorylation [11] to activate CRE-mediated *period 1–2* transcription [12,13], thereby setting the phase of the clock.

Various drugs can modulate circadian entrainment targeting NO• transduction, including organic nitroso derivatives such as S-Nitroso-N-acetylpenicillamine (SNAP, an S-nitrosothiol), or *N*-nitrosomelatonin (NOMel), both acting as NO• donors [14,15]. While NO• generation by NOMel at the SCN only potentiates photic phase-advances—and doesn’t delay even at high doses [15]—administration of SNAP potentiates both photic delays and advances [14]. At high doses SNAP can work as an NO• donor, increasing cGMP, but low doses generate S-nitrosation with opposite effects in cardiomiocytes [16]. On the other hand, the nNOS antagonist N-nitro-L-arginine methyl ester L-NAME attenuates both phase-advances and phase-delays of locomotor activity rhythms in hamsters [14,17]. Extracellular scavenging of NO• with 2-Phenyl-4,4,5,5-tetramethylimidazoline-1-oxyl 3-oxide (PTIO) blocks photic phase-advances, but not delays, which demonstrates its role as a paracrine messenger, by decreasing ventral-dorsal communication of light message at the SCN [18].

A hypothesis for circadian pathway bifurcation downstream of NO• [19] states that the photic activation of sGC hemoprotein occurs with diffusible, long-range (i.e., far from its nNOS source) NO• concentrations [20,21], which, despite being a highly diffusible, membrane-permeable gaseous messenger, participates in an array of redox-dependent, secondary reactive nitrogen species, as S-nitrosothiols (i.e., S-nitrosated glutathione, GSNO), nitroxyl anion (NO^−^), and metalnitrosyl complexes [22]. In addition, redox forms of NO• may act in so-called redox-dependent signaling to form S-nitrosation (i.e., the addition of a nitroso group by condensation of a thiol and the one-electron oxidation of NO•, nitrous acid) [20,23]. The one-electron reduced (and protonated at physiological pH) relative of NO• nitroxyl (HNO, azanone, nitrosylhydride, among other names) can be generated by a number of biological processes: HNO is a by-product of S-nitrosothiols reaction with cysteine thiols [24] and is even produced by nNOS [25]. Pharmacologically, it can be generated by the decomposition of sodium trioxodinitrate (Na_2_[N_2_O_3_] Angeli’s salt, AS) together with sodium nitrite at physiological conditions [26]. HNO is widely studied in the cardiovascular system [27], and can be easily oxidized to NO• at physiological conditions (but not the reciprocal reduction of NO•) [28]. However, NO• and nitroxyl exhibit differential reactivity toward metalloproteins, as well as different thiophilicity depending on redox intermediates, which might explain their distinctive physiological roles [28]. Moreover, while NO• increases cGMP through sGC, HNO activates a calcitonin gene-related peptide [29], increasing cAMP, and also modulates calcium channels and ryanodine receptors [30]. Thus, HNO is proposed as a signaling molecule with more distinctive effects than NO• and is thus worthy of continued study regarding its role in circadian entrainment.

In this context, we investigated the role of NO• relative nitroxyl in the circadian clock entrainment under pharmacological administration of Angeli’s Salt, a diazeniumdiolate-based nitroxyl donor. In addition, the antioxidants N-acetylcysteine (NAC) and glutathione (GSH) were used to test whether the pharmacological modulation of the SCN-redox environment can affect circadian entrainment. In this way, we provide evidence of the role of putative redox forms of NO•, as well as the redox modulation of SCN, driving this key circadian function.

## 2. Results

### 2.1. Effect of the Nitroxyl Donor Angeli’s Salt on Phase-Shifts of Locomotor Rhythms

**Figure 1:**
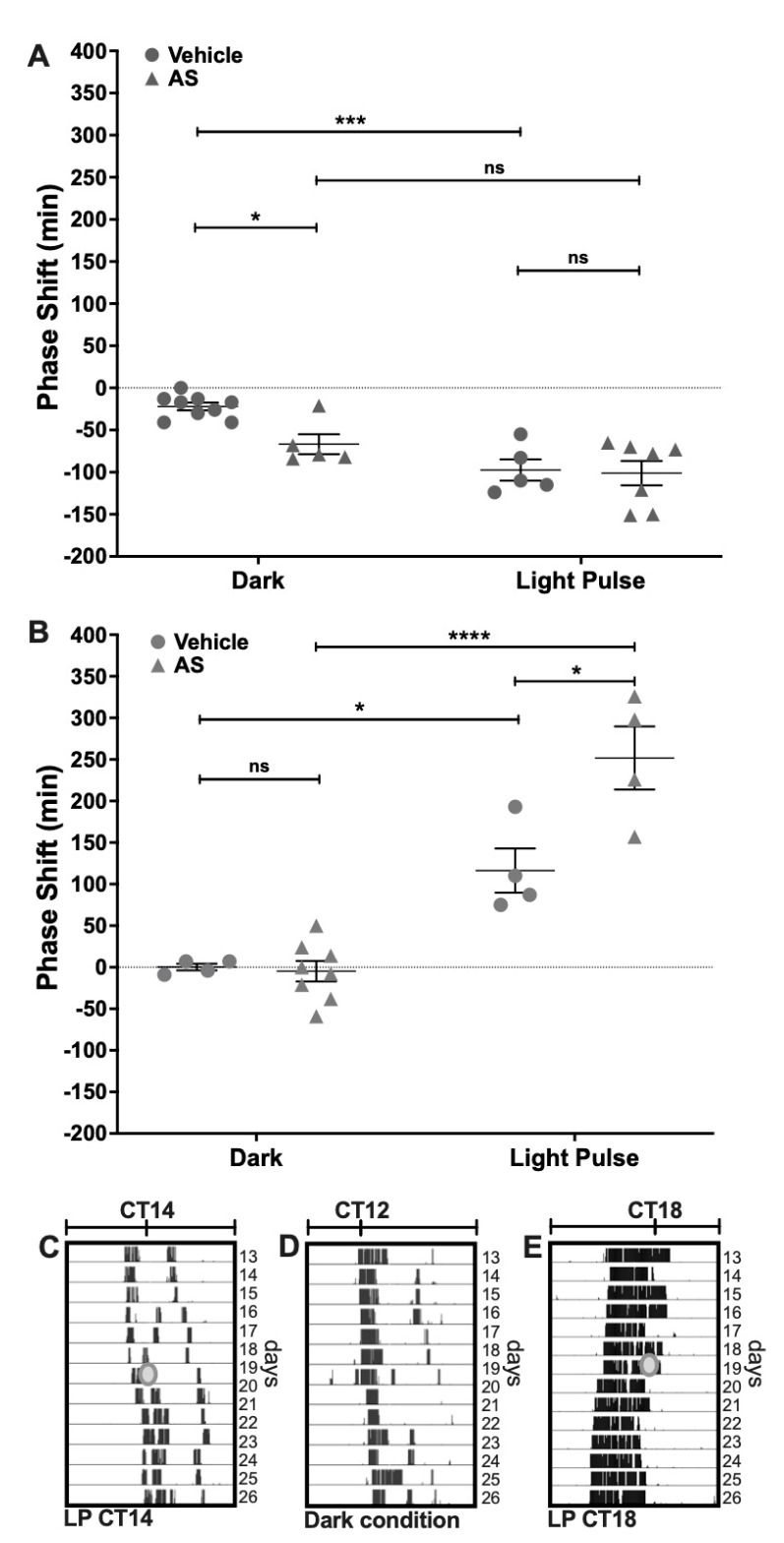
Effect of the nitroxyl donor AS on phase changes induced by light. Hamsters in constant darkness conditions, treated with an i.c.v injection of vehicle or 100 µM AS, 5 min before a subsaturant light pulse (LP, 10 min, 50 lux) at two CTs: (**A**) CT14 and (**B**) CT18. Control animals (Dark) received only pharmacological treatment without the light pulse. Phase changes are shown as mean ± SEM. Two-way ANOVA, followed by Tukey’s multiple comparisons test, ns = not significant; * *p* < 0.05; *** *p* < 0.001; **** *p* < 0.0001. CT14 Dark: Veh *n* = 9, AS *n* = 5; CT14 LP: AS + LP *n* = 7, Veh + PL; CT18 Dark: Veh *n* = 4, AS *n* = 7; CT18 LP: Veh + LP and AS + LP *n* = 5. (**C**–**E**) are representative actograms showing: (**C**) the effect of a light pulse at CT14, inducing a phase-delay in the circadian locomotor rhythms; (**D**) the phase of an animal without light stimulation during the subjective night; and (**E**) the effect of light stimulation at CT18, which induces a phase-advance. The grey circle indicates the day and time of the LP.

Phase-shifting experiments under DD conditions were performed to assess the effect of Angeli’s Salt (AS) administration on photic entrainment. AS, a well-known nitroxyl donor, was delivered i.c.v. (1 μL, 100 μM) 5 min before a subsaturant LP at CT14 (to induce delays) or CT18 (for advances). We found a circadian-dependent effect of AS over circadian phase-shifting. At CT14, this nitroxyl donor induced a phase-delay when administered alone in the dark, as compared with the vehicle (AS dark: 66.8 min ± 26.3 min, *n* = 5; Vehicle dark: 22.0 min ± 13.7 min, *n* = 9; *p* < 0.05 Tukey’s multiple comparisons test) (Figure 1A). AS did not affect light-induced phase-delays at CT14 (AS LP: 101.1 min ± 38.4 min, *n* = 7; Vehicle LP: 97.4 min ± 28.2 min, *n* = 5, respectively; *p* = ns Tukey’s multiple comparisons test) (Two-way ANOVA; LP *p* < 0.0001, F = 24.82, DFn = 1, DFd = 22; Drug *p* > 0.05, F = 4.85; interaction *p* = ns, F = 3.47) (Figure 1A). On the other hand, at CT18 AS administration potentiated the effect of light, as it significantly increased phase-advances as compared with the vehicle (AS LP: 251.7 min ± 75.9 min, *n* = 4; Vehicle LP: 116.2 min ± 53.2 min, *n* = 4, respectively; *p* > 0.001 Tukey’s multiple comparisons test) (Figure 1B), but did not generate any effect on the circadian phase when delivered alone (AS Dark: 0.3 min ± 8.1 min, *n* = 4; Vehicle Dark: 4.7 min ± 34.9 min, *n* = 8, respectively; *p* = ns Tukey’s multiple comparisons test) (Two-way ANOVA; LP *p* < 0.0001, F = 73.55, DFn = 1, DFd = 16; Drug *p* > 0.01, F = 9.03; interaction *p* < 0.01, F = 10.46). These results indicate that AS modulates light-induced phase-shifts at CT18 but has no effect on light signaling at CT14 (i.e., AS modulates light-induced phase-advances but not light-induced phase-delays). On the other hand, AS administration at CT14, without LP, has a photic-like effect—inducing phase-delays of the circadian rhythm.

### 2.2. Electrochemical Detection of Nitroxyl (HNO) In Vivo at the SCN

**Figure 2:**
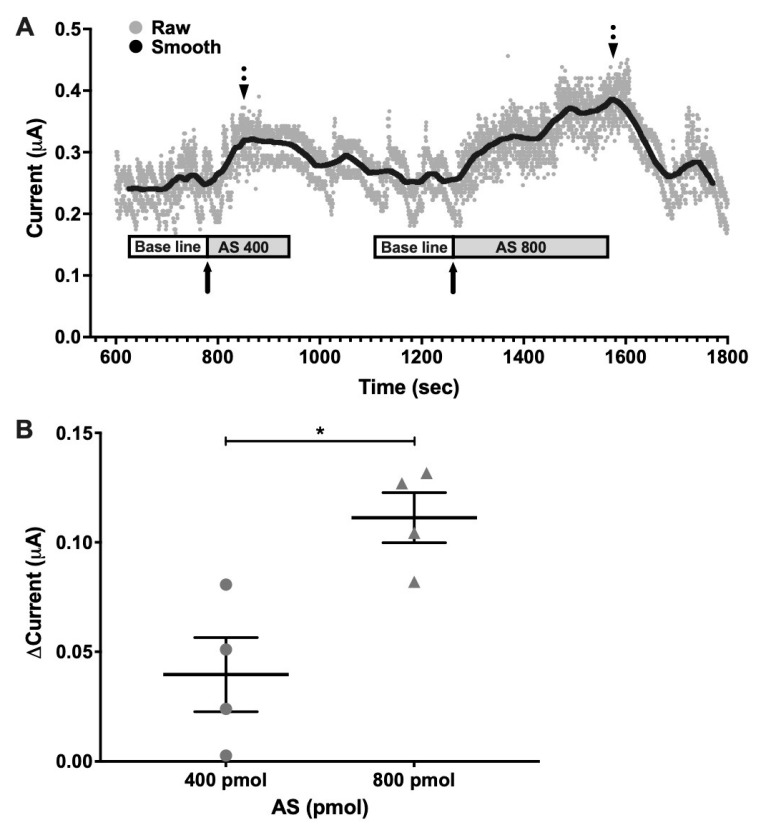
Electrochemical detection of nitroxyl (HNO) in vivo at the SCN. HNO generated in vivo by decomposition of Angeli’s Salt (AS) donor detected by time-resolved electrochemical quantification at the SCN in the pmolar range. (**A**) Representative graph of individual raw (Raw) and filtered (Smooth) data series using a moving average with a step of 300 samples to obtain 1 min-bin. After at least 2 min of baseline current register (white boxes), AS was delivered i.c.v from a 100 µM solution at 1.5 µL/min rate (grey boxes). Filled arrows indicate the time of injection with 400 pmol and 800 pmol of AS which corresponds with the registration of the baseline current, while dotted arrows indicate maximum current, which correlates to maximum HNO production. (**B**) Delta current values, calculated as the difference between maximum post-injection and basal prior-injection current values, for 400 pmol and 800 pmol AS. *n* = 4; * *p* < 0.05, paired *t-*test.

Decomposition of AS is known to produce both HNO and NO• [31,32]. To verify if HNO was effectively generated at physiological conditions, time-resolved electrochemical quantification was performed in vivo at the SCN in anesthetized hamsters. Raw data series showed changes in current intensity measured in a representative individual for detecting HNO concentration at the SCN. Smoothed data allowed paired measures at baseline (vehicle-treated) and after AS microinjection in the same individual (Figure 2A). Differences in current intensity were calculated as the difference between maximum current values after AS treatment and baseline values. Current intensity (μAmperes, μA) showed increased values after both 400 pmol and 800 pmol doses. A significant difference in this current increment was found regarding AS concentration (Figure 2B), showing higher increment for 800 pmol (0.111 μA ± 0.022 μA) as compared with 400 pmol (0.039 μA ± 0.033 μA) (*p* < 0.05 paired Student’s *t-*test; *t* = 5.14; df = 3), indicating HNO generation by AS in a dose-dependent manner. Using a similar concentration order (400 and 800 pmol) as that used for phase/shifting experiments (100 pmol) these results allowed us to verify that HNO is pharmacologically generated by decomposition of AS after i.c.v. delivery of the drug in the SCN.

### 2.3. Measurement of the Redox Pair GSH–GSSG at the SCN

**Figure 3:**
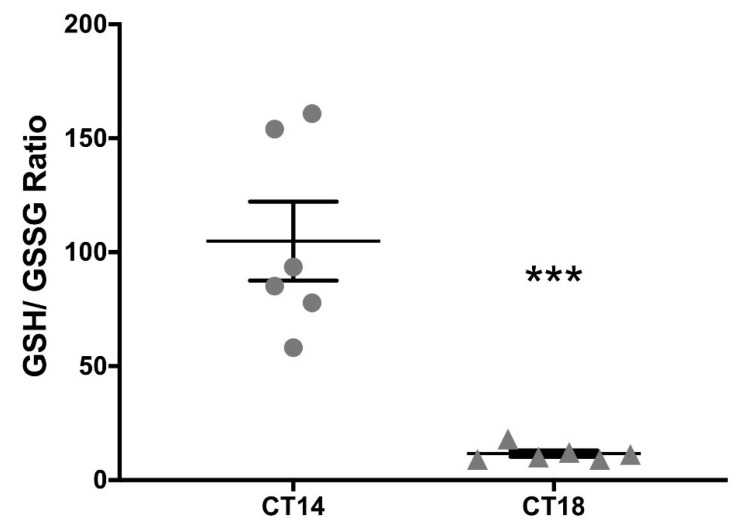
Glutathione couple measurement in the SCN at subjective night. The redox state was assessed as the GSH/GSSG ratio. Brains were collected at CT14 and CT18 and samples were frozen at −80 °C. SCNs from 2–3 individuals were pooled for measuring reduced (GSH) and total glutathione, inferring oxidized glutathione (GSSG) values using the equation GSSG = (total glutathione—GSH)/2. The rate relation was also calculated as GSH/GSSG, thus indicating a more oxidized environment when the relation was lower. Significantly lower values for CT18 were found, indicating a more oxidized environment at late subjective night (CT14: 104.9 μm/(mg/mL) ± 42.38 μm/(mg/mL) vs. CT18: 11.62 μm/(mg/mL) ± 3.37μm/(mg/mL)) (*** *p* < 0.001 paired *t-*test; *t* = 5.38; df = 10; *n* = 6).

Since HNO generated by AS may be favored under a reductive environment [33], the GSH/GSSG couple was measured at CT14 and CT18 in SCN tissue homogenates as a reliable measure of the cellular redox state [34]. We found a significant difference in total GSH between CT14 and CT18 (10.29 μm/(mg/mL) ± 3.99 μm/(mg/mL) vs. 16.42 μm/(mg/mL) ± 6.89 μm/(mg/mL), respectively) (*p* < 0.05 paired Student’s *t-*test; *t* = 2.43; df = 18; *n* = 6). When GSH was analyzed, we found no significant differences (CT14: 8.73 μm/(mg/mL) ± 3.30 μm/(mg/mL) vs. CT18: 9.98 μm/(mg/mL) ± 3.91 μm/(mg/mL)). Nevertheless, GSSG was significantly higher at CT18 when compared with CT14 (CT14: 0.13 μm/(mg/mL) ± 0.11 μm/(mg/mL) vs. CT18: 0.58 μm/(mg/mL) ± 0.31 μm/(mg/mL)) (*p* < 0.01 paired *t* test; *t* = 3.99; df = 15; *n* = 6), indicating a more oxidized state at CT18 in the SCN. This was confirmed when analyzing the GSH/GSSG ratio, resulting in significantly lower values for CT18 (CT14: 104.9 μm/(mg/mL) ± 42.38 μm/(mg/mL) vs. CT18: 11.62 μm/(mg/mL) ± 3.37 μm/(mg/mL)) (*p* < 0.001 paired *t-*test; *t* = 5.38; df = 10; *n* = 6) (Figure 3). These results allowed us to hypothesize that pharmacological generation of HNO could be favored under a reductive environment at CT14.

### 2.4. Effect of NAC and GSH Administration on Light-Induced Phase-Shifts

**Figure 4:**
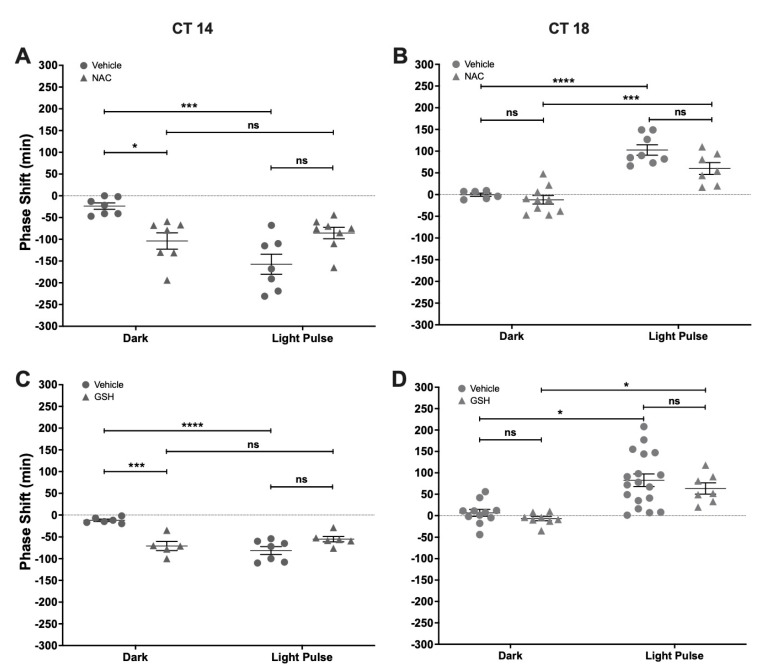
Effect of antioxidants N-acetylcysteine (NAC) and glutathione (GSH) on circadian phase changes. Groups of hamsters in constant darkness conditions were treated with an i.c.v injection of either NAC (2 µL, 100 µM, or vehicle) (**A**,**B**) or GSH (1 µL, 100 µM) (**C**,**D**), 5 min before a subsaturant light pulse (10 min, 50 lux) delivered at CT14 or CT18. Controls for photic response (Dark) received only pharmacological treatment without light stimuli. Phase changes are shown as mean ± SEM. Two-way ANOVA, followed by Tukey’s multiple comparisons test, ns = not significant; * *p* < 0.05; *** *p* < 0.001; **** *p* < 0.0001; for NAC experiment at CT14: vehicle Dark *n* = 7, vehicle LP *n* = 8, NAC Dark *n* = 7, NAC LP *n* = 8; at CT18: vehicle Dark *n* = 6, vehicle LP *n* = 8, NAC Dark *n* = 10, NAC LP *n* = 7. For GSH experiment at CT14: vehicle Dark *n* = 6, vehicle LP *n* = 7, GSH Dark *n* = 5, GSH LP *n* = 6; at CT18: vehicle Dark *n* = 11, vehicle LP *n* = 17, GSH Dark *n* = 8, GSH LP *n* = 7).

To test the effect of changing the redox environment on photic entrainment, we performed a set of experiments that used acute i.c.v. treatment with NAC or GSH, paired with a subsaturant LP. When compared with vehicles, the photically-induced shifts were not affected by NAC, both at CT14 or CT18 (Figure 4A,B). Also, NAC delayed the phase of locomotor rhythm at CT14 in a magnitude that was significantly different from the vehicle (Vehicle Dark: −23.71 min ± 19.54 min; NAC Dark: −104 min ± 49.7 min) but did not affect light-induced phase-delays (Vehicle LP: −157.4 min ± 61.22 min; NAC LP: −85.63 min ± 37.29 min) (Two-way ANOVA; LP *p* < 0.001, F = 21.17, DFn = 1, DFd = 25; Drug *p* = ns, F = 0.06; interaction *p* < 0.001, F = 21.17). NAC had no effect over circadian synchronization at CT18 by itself (Vehicle Dark: −0.33 min ± 9.16 min; NAC Dark: −12 min ± 30.87 min) or with a concomitant light pulse (Vehicle LP: 102.6 min ± 33.83 min; NAC LP: 60 min ± 36.14 min) (Two-way ANOVA; LP *p* < 0.0001, F = 62.23, DFn = 1, DFd = 27; Drug *p* < 0.05, F = 5.99; interaction *p* = ns, F = 1.95). A similar result was found for GSH, which induced phase-delays at CT14 (Vehicle Dark: −12.17 min ± 6.67 min; GSH Dark: −70.8 min ± 23.38 min) and did not change the effect of light pulses (Vehicle LP: −81.29 min ± 23.94 min; GSH LP: −55 min ± 15.186 min) (Two-way ANOVA; LP *p* < 0.01, F = 30.47, DFn = 1, DFd = 20; Drug *p* < 0.05, F = 12.01; interaction *p* < 0.0001, F = 30.47) (Figure 4C). Also, GSH had no effect at CT18 either by itself (Vehicle Dark: 6.45 min ± 26.77 min; GSH Dark: −7 min ± 13.96 min) or interacting with light (Vehicle LP: 82.72 min ± 62.37 min; GSH LP: 63.43 min ± 34.64 min) (Two-way ANOVA; LP *p* < 0.0001, F = 25.42, DFn = 1, DFd = 40; Drug *p* = ns, F = 1.27; interaction *p* = ns, F = 1.27) (Figure 4D). These results show that antioxidants NAC and GSH are able to modulate the circadian phase at CT14.

## 3. Discussion

Evidence pointing to NO• as a diffusible, intra and extracellular messenger participating in the photic transduction of circadian phase-advances is based on: (1) the increased activity in the nNOs/sGC/GMP/PKGII pathway after a light-pulse at CT18 [7,35,36,37]; (2) the indirect pharmacological reduction of phase-advances at CT18 by nNOS [14,15,17], sGC, and PKGII inhibition [35]; (3) scavenging of extracellular NO• [18]; and (4) enhancement of photic-advances by NO• donors as SNAP [14] and N-nitrosomelatonin [15]. Taken together, the current evidence indicates that a substantial increase in intra and extracellular NO• by its light-induced synthesis at the SCN should be expected at CT18 for phase-advances, as compared with phase-delays.

Here we assessed the role of HNO, a redox form of NO•, in its ability to modulate photic entrainment in the hamster SCN. This compound had two pharmacological effects: it generated phase-delays when delivered alone, and potentiated photic phase-advances. HNO was electrochemically measured in a dose-dependent relationship in the hamster SCN after delivering the AS donor, verifying its ability to increase HNO in vivo. The measurement of the GSH/GSSG ratio estimates SCN, GSH, and GSSG concentrations in a 1–10 mM range [38]. We found a reductive environment at CT14, which may favor the action of the HNO donor AS to generate phase-delays. Rate constants point that HNO oxidation is rather specific for thiols as reductants, while minor for nitrogenous species such as amines, indoles, and purines [29]. HNO oxidation should be determined by the availability of major cellular free thiols, such as glutathione, under reductive environment [20], while an oxidative environment would induce oxidation to NO• and auto-dimerization and/or reaction with oxygen to form nitrous oxide, nitrate, nitrite [28,39], and peroxynitrite [40]. Moreover, physiological detection of NO• by nNOS synthesis is dependent on oxidation of HNO (a primary product of nNOS) to NO•, catalyzed by the increase in superoxide dismutase activity under an oxidative environment [41], although this finding I challenged by [42]. Also, HNO can directly increase sGC enzymatic activity *per se* as found in purified lung extracts [43].

Biological actions of HNO are largely explained due to its reactivity to free thiols (i.e., GSH) and cysteine residues. Most studies assessed its effects in the cardiovascular system, associated with the oxidation of free cysteine thiol residues [30], as well as with sarcoplasmic RyRs, increasing calcium release [44]. A few studies were done in the central nervous system, comparing the effects of AS with the NO• donor diethylammonium (Z)-1-(N,N-diethylamino)diazen-1-ium-1,2-diolate (DEA/NO) in the hamster retina [45]. Compared with DEA/NO, AS generates similar inhibition of nNOS activity and L-citrulline transport and reduction of GSH and protein S-nitrosation, but less cGMP induction. High (400 nM), chronic (7 days) AS, administered intranigrostriatally as a model of neurodegeneration, induces GSH depletion and dopaminergic neuronal loss [46]. The relatively high GSH concentrations we measured at CT14 (1–10 mM) is not expected to change after reacting with low HNO concentration generated by 100 pmol AS in the SCN. At low HNO concentration, reaction with thiol proteins to form S-nitrosation is feasible without altering free thiol concentration [28], via thiol condensation with the one-electron oxidation of NO•, nitrous acid [20,23], or via dinitrosyl iron complexes [47]. In addition, to separate both pathways based only on the global redox state (favoring the action of HNO at CT14) is a simplification, since in fact S-nitrosation should be favored under an oxidative environment; indeed, other factors such as target availability must be considered.

Although the SCN is very robust in keeping the circadian phase, here we report that both antioxidants GSH and NAC generate phase-delays of locomotor rhythms at CT14. In pathologies involving oxidative stress, NAC has several possible mechanisms of action as a therapeutic antioxidant: it can (1) act as a free-radical (RNS and ROS) and reactive electrophile scavenger; (2) increase the synthesis of GSH by its rapid deacetylation in the cell to form cysteine-cystine; and (3) reduce disulfide bonds [48]. We found similar circadian effects of GSH and NAC. In other models, NAC has been reported to deliver cysteine-cystine increasing GSH in the brain [49] for long term treatment as needed in neurodegeneration [50]; however, our results suggest a faster activity that should happen within a 3-h time-window in order to induce circadian resetting through changes in *per1–2* activity. In addition, it was shown that the inhibition of the activity of nNOS/cGC/PKGII input pathway components [36,37] delayed the circadian phase, so we could hypothesize that the phase-delays induced by GSH or NAC could be generated by a redox-dependent inhibition of the same pathway, as occurs with NOS activity and L-arginine uptake by AS treatment in the retina [45]. While Wang et al. [5] evidenced the effect of GSH on resting electrical activity in SCN slices, few circadian studies were done with NAC, which had inconsistent effects [51,52].

Measuring glutathione couple (GSH/GSSG ratio) in the SCN indicated a higher reductive environment at CT14, when compared with CT18. A recent study also showed high GSH concentration in rat brain tissue during the day [53]. In rat SCN organotypic slices, a relatively oxidized state at CT14 was reported by measuring glutathiolation [5] (i.e., the addition of glutathione to protein thiols via oxidative intermediates GSSG and/or GSNO, [54]), and dehydroascorbic/ascorbic acid ratio. The methods used in this study differ from the ones reported here, since the authors sacrificed rats and recorded redox states in sliced brain. Our results do agree with others in which the redox state was derived from glutathiolation state at CT6 vs. CT14 in SCN homogenates [55]. Experiments done by Wang et al. [5] were the first to assess a circadian rhythm in SCN redox state, coupled with the rhythm in membrane excitability by post-translational redox regulation of both leak and A-type K^+^ channels.

Several post-translational inputs from metabolic and redox pathways regulate the activity and stability of core clock proteins, such as heme binding on REV-ERBa and REV-ERBb, regulating BMAL1 stability and repression [10], or the interplay between zinc binding and disulfide bond formation, stabilizing the PER2:CRY1 heterodimer [56]. Increasing the oxidative environment by acute treatment with hydrogen peroxide was shown to destabilize PER2, altering the phase and amplitude of PER2: LUC bioluminescence rhythms in fibroblasts [57]. Indeed, a circadian oscillation of hydrogen peroxide determines a redox oscillation of CLOCK through Cys195 thiol oxidation, which promotes its interaction with BMAL1, enhances transcriptional activity of clock genes, and sets the molecular oscillator period in vitro [58].

To conclude, our results support the idea that the redox state could modulate SCN clock messengers for entrainment pathways downstream of NO•, as well as work as an input for circadian phase determination. Delivering nitroxyl by AS at the SCN exposes this redox-dependent form of NO• as a noncanonical messenger in the control of the circadian phase. The putative targets for nitroxyl in the SCN—as well as the redox factors affecting the circadian clock—remain to be established, whereas S-nitrosation of ryanodine receptors and/or clock proteins emerge as strong candidates for involvement in this modulation.

## 4. Materials and Methods

### 4.1. Pharmacological Modulation of Photic Entrainment

#### 4.1.1. Animals and Housing

Male, 3–4-month-old Syrian hamsters (*Mesocricetus auratus*) (Laboratorio Azul Diagnóstico, Azul, Buenos Aires, Argentina) were used. Animals were put in group cages of 5 individuals and acclimatized in stock rooms for at least two weeks under standardized conditions (14 h light: 10 h dark (LD 14:10), 20 (±2) °C), before entering the experimental protocols. Light was set as 200 lux of white “cool” light at cage bedding level. Lights-OFF was defined as zeitgeber time 12 (ZT12). Animals received rodent diet chow (ACA Cooperación, San Nicolás, Buenos Aires, Argentina) and tap water *ad-libitum*, with wood chip bedding renewal every 4 days.

#### 4.1.2. Drugs

Reduced glutathione (GSH) and N-acetyl-L-cysteine (NAC) (Sigma Aldrich, St. Louis, MO, USA) were diluted from stock solutions. Sodium trioxodinitrate (Na_2_[N_2_O_3_] Angeli’s salt, AS) was synthesized at the laboratory of Fabio Doctorovich (INQUIMAE-CONICET, Universidad de Buenos Aires) as described in [59]. Fresh drug dilutions were used just before administration.

#### 4.1.3. Surgeries and Intracerebroventricular Microinjections

For intracerebroventricular (i.c.v.) administrations, a 22-gauge steel guide cannula (Plastic Products, Roanoke, VA, USA) was implanted under isoflurane (5% in oxygen for induction, 2% for maintenance, 200 mL/min) aimed at 1.0 mm above the target site between the bilateral SCN (stereotaxic coordinates: anterior/posterior = +0.6, medial/lateral = 0.0, dorsal/ventral = −8.0 mm from bregma). After surgery, hamsters were kept under standard 14:10 LD cycles to recover for 7 to 10 days before being transferred to constant darkness (DD) for the duration of the experiment. All microinjections were performed under darkness in agreement with DD conditions (i.e., CT14 and CT18) with the use of a dim red light to aim and gentle restraint of the animals for about 1 min to deliver 1 μL using a Hamilton microsyringe (Reno, NV, USA). All animal procedures were performed in accordance with the Institutional Animal Care and Use Committee of the Universidad Nacional de Quilmes.

#### 4.1.4. Behavioral Experiments

The locomotor activity rhythm was obtained to assess the SCN clock output response under typical light-pulse experiments (i.e., nonparametric entrainment paradigm) (Figure 1, panels C–E) (see Statistical and Chronobiological Analyses, below). Wheel running activity was recorded using a data acquisition system designed in our lab. Hamsters were transferred into light-isolated closets with individual cages equipped with a running wheel to obtain time series of wheel revolutions accumulated, recorded each 5 min to a hard drive. For light-pulse (LP) experiments, the design includes two treatments as factors in an unpaired design: light induction (LP, or dark), and drug (drug, or vehicle VEH). The variable analyzed was the phase-shift, which was assessed in individual actograms (see Statistical and Chronobiological Analyses). Activity onset was eye-fitted in actograms as a typical circadian phase marker, defined as circadian time 12 (CT12). The behavioral protocols were as follows: hamsters were transferred from LD 14:10 to constant darkness (DD) for at least 15 days to obtain a stable free-running period under DD conditions. Then, a subsaturant LP (50 lux, 10 min) was delivered at two experimental CTs: CT14, to induce phase-delays, and at CT18, to induce advances. Drug delivery was set 5 min before the LP, in subgroups of hamsters that were separated to receive an i.c.v. injection of: AS (1 μL, 100 μM), GSH (1 μL, 100 μM), or NAC (2 μL, 100 μM). For the duration of the protocols, animals received food and tap water ad libitum, and cages were cleaned and bedding was replaced every 7 days.

#### 4.1.5. Electrochemical Detection of Nitroxyl In Vivo at the SCN

Nitroxyl generated in vivo by Angeli’s Salt donor was detected at the SCN in the pmolar range by time-resolved electrochemical quantification [60]. To this end, hamsters under LD cycles were implanted under darkness at ZT14 with the sensing electrode coupled to an internal cannula at SCN stereotaxic coordinates (see above in Surgeries and Intracerebroventricular Microinjections) for acute infusions (1.5 µL/min). Current intensity generated was monitored in real-time and registered each 0.2 s during the course of the experiment. Once steady baseline recording was set under vehicle infusion, paired drug-treatments were tested in each subject (using sequential microinjections of 2, 400, and 800 pmol; see Figure 2) with vehicle wash-out prior to all drug administrations (200 pmol dosing did not generate evident changes in the signal, thus it was not included in the analyses). Individual raw data series were filtered for high-frequency noise using a moving average with a step of 300 samples to obtain 1 min-bin series for the following calculations: two time-windows of 3 min data were averaged, one to set baseline values, taking 3 data points prior to drug injection, and the other post-drug infusion to set maximum, taking 3 data points around the higher recorded value. Maximum and baseline were subtracted to calculate the change in the electrical current.

#### 4.1.6. Measurement of the Redox Pair GSH/GSSG at the SCN

To study the redox state of the SCN at subjective night, we focused on the pair GSH–GSSG, the main redox couple in the cell [38]. Hamsters were kept under DD conditions and groups were set at CT14 or CT18. Individuals were euthanized by decapitation and their brains frozen in liquid nitrogen. For determination of GSH–GSSG levels, a commercial kit (Fluorometric—Green, Abcam, Cambridge, UK) with maximal detection of 10 μM was used following the provider’s instructions. Briefly, a 1-mm thick coronal slice containing the SCN was sectioned from the brain. A tissue sample was extracted just above the chiasm at the suprachiasmatic region. Samples corresponding to 2–3 brains were pooled (i.e., *n* = 1 corresponds to 2–3 pooled brains of the same experimental group) for each determination of GSH and total glutathione (GSH + GSSG) values, inferring GSSG values using the equation GSSG = (total glutathione − GSH)/2. Values obtained were relativized in proportion to the total amount of protein, measured by Bradford Assay. Measurements were performed using a Cytation 5 Imaging reader (Biotek Instruments, Winuschi, VT, USA), at Ex/Em = 490/520 nm. The GSH/GSSG ratio was calculated, indicating a more oxidized environment when the value is lower.

#### 4.1.7. Statistical and Chronobiological Analyses

Individuals were randomly assigned for all the experiments into their corresponding groups. For the GSH/GSSG ratio experiment, each experimental sample is composed of a pool of SCN tissue obtained from 2–3 hamster brains. Normal distributions were tested with Shapiro–Wilk normality test and then parametric analyses were performed (*t*-test, one-way and two-way ANOVA, as indicated in the text) and post-hoc tests (Bonferroni and Tukey’s multiple comparison tests) were applied when indicated. Results are presented as mean ± SEM of experimental datasets. Statistical analyses were performed with GraphPad Prism 8 (GraphPad Software, La Jolla, CA, USA) software. *p*-values lower than 0.05 were considered to reach statistical significance. Time-series of wheel running activity were represented in actograms plotted at modulo Tau (i.e., using the free running period of the corresponding individual) to analyze the phase of locomotor rhythms (panels C–E in the Figure 1 show actograms of representative rhythms). An eye-fitted line set at the onsets of locomotor activity were obtained in the actograms as a typical marker of the circadian phase, to obtain the times for CT12 and CT18. Phase-shifts were calculated by fitting a line through activity onsets 15 days prior to, and between 5 and 15 days after, the day of treatment; the time difference between the extrapolation of these two lines on the day of treatment (i.e., a phase-shift) was calculated. Chronobiological analyses were done using an integrated package for chronobiology, El Temps (Antoni Díez Noguera, Universitat de Barcelona, Spain).

## Data Availability

The datasets used and/or analyzed during the current study are available from the corresponding author on reasonable request.

## Sample Availability

Samples of the compounds are available from the corresponding author on reasonable request.
